# Cafeteria-Type Feeding of Chickens Indicates a Preference for Insect (*Tenebrio molitor*) Larvae Meal

**DOI:** 10.3390/ani10040627

**Published:** 2020-04-06

**Authors:** Marcos Antonio Nascimento Filho, Raquel Tatiane Pereira, Ana Beatriz Santos de Oliveira, Diana Suckeveris, Alvaro Mario Burin Junior, Thiago de Araújo Mastrangelo, Diego Vicente da Costa, José Fernando Machado Menten

**Affiliations:** 1 Department of Animal Science, University of São Paulo, Piracicaba, SP 13418-900, Braziljfmenten@usp.br (J.F.M.M.); 2 Radioentomology and Food Irradiation Laboratory, Center of Nuclear Energy in Agriculture, Piracicaba, SP 13416-000, Brazil; 3 Agricultural Sciences Institute, Federal University of Minas Gerais, Montes Claros, MG 39404-547, Brazil

**Keywords:** insect meal, *Tenebrio molitor*, broiler, alternative ingredient, acceptability, nutritional value, performance

## Abstract

**Simple Summary:**

The use of insects as an alternative ingredient in the feed industry is a promising solution to optimize animal production systems worldwide. These insect-derived products are seen as novel sources of animal origin protein, especially in avian and aquatic species diets, which are sustainable in production and desirable as nutrient-rich feed ingredients. In order to be used in feed formulations for poultry, the nutritional composition of the insect products and the effects on performance of chickens must be known. In the present study, we investigated whether broilers displayed a preference (or not) for *Tenebrio molitor* larvae meal, evaluating ingredient acceptability and birds’ performance. After a few days of being offered insect meal in a cafeteria-type study, chickens developed a clear preference for this ingredient compared to usual feed ingredients, especially extruded semi-whole soybean meal (high protein content). Additionally, there was an indication that *T. molitor* meal consumption by the chickens improved feed conversion. We conclude that *T. molitor* meal is a promising protein ingredient for poultry diets. Overall, although insect-derived products are still under regulation processes all around the world, the increasing knowledge concerning this topic indicates that insects could be a suitable alternative as feed source in the animal industry.

**Abstract:**

This study aimed to determine whether broiler chickens display a preference for *Tenebrio molitor* larvae (TM) meal by evaluating ingredient acceptability and birds’ performance. Sixty 14-day-old male chickens were assigned into two treatment groups (5 birds/pen, *n* = 6) in a cafeteria-type study: the control (C) group, and the TM group. Each pen was equipped with one bell drinker and four through feeders allocated side by side; all feeders of the C group contained a complete standard diet whereas each feeder of the TM group contained one of the following ingredients: ground corn, extruded semi-whole soybean, vitamin-mineral supplement mixture, and TM meal. Feed intake was recorded daily and growth was monitored periodically up to day 32. Chickens which had access to individual feed components showed a delay to display preference for TM, but consumed, overall, up to 50% of the total intake as TM meal. Feed intake and growth performance were lower in all periods for TM group (*p* < 0.02), whereas feed conversion ratio was improved on days 22–28 and days 29–32 of age (*p* < 0.01). Data from bivariate and multidimensional analysis indicate that birds started to reach a balance of ingredient intake at 25 days of age, showing a high correlation between consumption of each ingredient and the day of the experiment. Chickens exhibited a preference for *T. molitor* meal, resulting in improved feed efficiency, which allows us to conclude that it can be a suitable feed alternative for poultry.

## 1. Introduction

The use of insects in animal nutrition is a promising alternative in order to obtain a sustainable protein source to feed the world. Considering the current challenges of overpopulation and feed supply for animals and humans, new feed ingredients are needed to provide a secure food production chain in the future [1].

Edible insects have been shown to be highly nutritious and healthy food sources (rich in protein and fat), with beneficial nutraceutical properties. Moreover, by seeking animal production systems that are more environmentally friendly, insect rearing has contributed positively to new sustainable ecosystems, requiring less water, food, space, and, most interesting, recycling organic by-products as substrate for growth [2,3,4].

Among circa 2000 species of known edible insects, *Tenebrio molitor* (TM) is one used to produce larvae meal for animal feeding [5,6]. The dried meal derived from TM larvae is rich in protein (47–60%) and fat (31–43%) content, and has been introduced in commercial pet and zoo animals’ diet [4,7].

Chickens have the natural behavior of picking up a variety of insects during their entire lifecycle and eating them voluntarily, and these insects may represent a part of the bird’s ingested food [3]. Moreover, studies have shown that birds are able to self-select available feedstuffs in order to balance their own diet, meeting nutritional requirements [8]. Considering that insect meal has a similar protein content to soybean meal, and soy cultivation requires vast arable land areas and leads to some environmental damage [9], it is feasible to suggest that insects can be introduced in feed formulation for chickens.

In addition to concerns about land usage, greenhouse gas emissions, public health, and water pollution [10,11], recent studies reveal how insect rearing systems can produce a beneficial food and feed source throughout the next years [12]. In 2017 the European Commission authorized the application of insect protein in aquaculture feed (EU 2017/893) [13], and it is expected that a new revision of the feed ban rules will allow insect protein in poultry and swine feed by 2020 [14]. In many countries there have been investments to support this alternative feed ingredient on the market for commercial-scale production, and animals might favor insects once they become a regular component of their diet [15].

Regarding its nutritional value, some studies have investigated amino acid profile, fatty acid content, nutrient digestibility, and health benefits of this alternative protein ingredient and demonstrated promising results, but the information is still limited and additional research is under development [16,17,18,19,20]. In order to provide new useful and accurate information on TM meal in practical diets for poultry, this study aimed to determine whether chickens display a preference for TM meal when offered simultaneously to corn, extruded semi-whole soybean, and supplement mixture, in a cafeteria-type trial, by evaluating ingredient acceptability and birds’ performance during the period from 14 to 32 days of age.

## 2. Materials and Methods 

The experimental procedures were approved by the Institutional Animal Care and Use Committee, University of São Paulo, Piracicaba, SP, Brazil (protocol number: 2017.5.2568.11.5; 17/11/2017).

### 2.1. Animals, Diets, and Experimental Procedures

A cafeteria-type feeding (free-choice) study was conducted at the Department of Animal Science, University of São Paulo, Piracicaba, São Paulo, Brazil. A total of 100 one-day-old male broiler chickens (individual body weight ~52 g) of a commercial strain (Ross AP95) were raised in floor pens (wood shavings as bedding material) and fed a corn-soybean meal starter diet. At day 14, 60 birds of uniform body weight (~459 g) were chosen and randomly distributed into two dietary treatments: a control (C) group, and a TM group (test group). Each pen was equipped with one bell drinker and four through feeders allocated side by side; all feeders of the C group contained a complete standard diet (Table 1) to meet birds’ nutritional requirements for standard performance [21], whereas each feeder of the TM group contained one of the following ingredients: ground corn, extruded semi-whole soybean, supplement mixture (vitamin–mineral premix, limestone, dicalcium phosphate, salt, choline chloride, amino acids, salinomycin), and TM meal. Each group consisted of six replicate floor pens (five birds/pen) assigned to a completely randomized design. The insect meal was obtained from Vida Proteína Cia. Ltd.a., Neirópolis, Goiás, Brazil. Feed and water were available ad libitum. All feeders were rotated of position daily to avoid eventual laterality of the animals. Supplement mixture was composed of one part of the mix of the minor components and three parts of sand in order to dilute and encourage consumption. The C group was used as a reference for total feed and nutrient consumption by the birds.

### 2.2. Measurements and Analytical Methods

Samples of TM meal, corn, extruded semi-whole soybean, and the standard diet were ground to pass through a 1-mm sieve and stored in plastic bags. Analyses were carried out to determine the dry matter (DM), ether extract (EE), and crude protein (CP). Additionally, ash, gross energy (GE), amino acid composition (AA), fatty acid profile (FA), calcium, phosphorus, copper, iron, manganese, and zinc content of TM meal were determined to characterize the ingredient. According to standard procedures proposed by Association of Official Analytical Chemists (AOAC) [22], the samples were dried to a constant weight at 105 °C for 24 h to determine the DM content (procedure 930.15). GE was measured using an oxygen bomb calorimeter (Parr 6200; Parr Instrument Co., Moline, IL, USA). Nitrogen was determined in order to calculate CP (N × 6.25) using AOAC [22] procedure 984.13, ash content using the furnace muffler at 550–600 °C, procedure 924.05, and EE by Soxhlet extraction method, procedure 920.39. Quantitative measurement of AA (except tryptophan) was performed by AMINOLab^®^ (Evonik Industries, Hanau, Germany) using a HPLC procedure with sample preparation by hydrolysis with the hydrochloric acid method for most amino acids, or by performic acid oxidation prior to the hydrolysis for methionine and cystine analysis [23] (procedure 994.12).

FA methyl esters (FAMEs) were analyzed using Focus gas chromatography (Thermo-Finnigan, San Jose, CA, USA) equipped with a flame ionization detector (FID) and a CP-Sil 88 capillary column (100 m length × 0.25 μm i.d. × 0.20 μm film thickness; Supelco, Bellefonte, PA, USA). The following temperature program was used: initial hold of 4 min at 70 °C; followed by rise at 13 °C/min to 175 °C and rise at 4 °C/min from 175 to 215 °C; and a final hold of 5 min followed by rise at 7 °C/min to 230 °C. The injector temperature was 250 °C. The injection volume was 1 μm. The detector temperature was 260 °C. Peaks were identified by comparison of retention times for known FAME standards with software (Chromquest 4.1, Thermo Electron, Monza, Italy) and FA contents were estimated by an area normalization method from Sigma as internal standard. The FA profile was expressed as % of total lipids. Mineral samples were determined by the CBO Laboratory (Campinas, São Paulo, Brazil) following AOAC [24] procedure method 927.02 for calcium, copper, iron, manganese, and zinc, and procedure method 965.17 for phosphorus. Similarly, corn, extruded semi-whole soybean, and the standard diet were analyzed for DM and CP, and the supplement mixture for calcium and phosphorus following the procedures mentioned above.

Broiler growth performance was measured starting on day 15 until 32 days of age. Due to the limited amount of insect meal available, the experiment was terminated when the supply of the product was finished. Feeders were weighed and refilled daily to determine the feed intake of each individual component per treatment pen. The consumption of sand used as an inert substance in the supplement mixture was not taken into account in the calculations. Birds were weighed on days 21, 28, and 32 to determine body weight gain and feed conversion ratio.

### 2.3. Statistical Analysis

Performance data were submitted to ANOVA by PROC GLM (General Linear Models) of SAS 9.4 [25]. When a significant effect was verified, the variables were submitted to mean comparison by *t* test within each evaluation period. For the test group, data of daily individual ingredient consumption were compared by Tukey test. In addition, these data of each feed ingredient in the six replicates of the test group were submitted to a parametric analysis (Pearson’s correlation coefficient) with descriptive statistics by PROC CORR of the SAS software to establish the day on which the intakes tended to plateau; in other words, when the consumption of TM meal was constant. In order to rate the preference of each ingredient consumed by the birds, a nonmetric multidimensional preference analysis (MDPREF) was performed through PROC PRINQUAL of the SAS program to identify whether or not there was preference for TM meal by the birds. When pertinent, data were evaluated considering the level of 5% of significance.

## 3. Results

The analyzed values for DM, EE, and CP were 868.9 g/kg, 29.6 g/kg, and 86.1 g/kg in corn and 931.1 g/kg, 125.3 g/kg, and 416.8 g/kg in extruded semi-whole soybean, respectively. The nutritional profile and mineral content of TM meal used in this study are summarized in Table 2 and compared to average values found in the literature. The total protein and fat content in TM larvae were 521 g/kg DM, and 317.4 g/kg DM, respectively. The GE content of TM meal on a dry matter basis was 28.45 MJ/kg. The mineral contents of TM meal were calcium (1228 mg/kg DM), phosphorus (6058 mg/kg DM), copper (6.8 mg/kg DM), iron (62.4 mg/kg DM), manganese (12.9 mg/kg DM), and zinc (115.1 mg/kg DM). For amino acid composition (Table 3), high values were found for valine (32.5 g/kg DM) and histidine (17.5 g/kg DM) in TM meal. Among the indispensable amino acids, leucine was the most abundant, whereas glutamic acid was the most abundant dispensable amino acid.

The fatty acid profile of TM meal is reported in Table 4. Concerning the main fatty acids in the test ingredient, significant amounts of palmitic, oleic, linoleic, and α-linolenic acid were observed, with values of 15.4, 45.3, 26.2, and 1.1 g/100 g of fat, respectively.

The results for daily average feed intake of each component offered to the birds in the six replicates for the test group are shown in Table 5. Up to day 17, corn was the ingredient consumed in greatest amount by the birds (*p* < 0.001). From day 18 until day 24, there was a shift in this trend and after that (day 25) the intake of TM meal was superior compared to all other components (*p* < 0.001). In Table 6 the Pearson’s correlation coefficient of feed intake of all ingredients between the ages of the birds at 23, 25, 27, 29, and 30 days of age is shown. Starting on day 25, there was a very high positive correlation (r = 0.93–0.98) among variables; on the other hand, r values for day 23 and the prior days of the experiment were lower (0.68–0.72), although significant.

**Figure 1:**
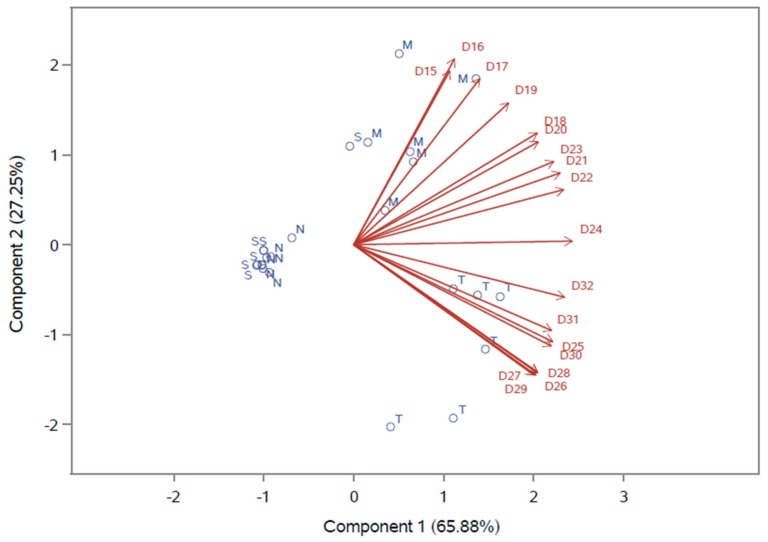
Biplot of multidimensional preference analysis for the consumption of test ingredients from day 15 to 32 of birds’ age (S = Extruded semi-whole soybean meal, N = Supplement mixture, M = Ground corn, T = TM meal).

In order to endorse the justification whether or not broilers have preference for TM meal or other food component in this study, a multivariate analysis graph for the feed consumption of the test group is presented in Figure 1. The graph shows a matrix containing the reference classification of the four components (represented as circles) for the 18 days of experimentation (represented as vectors). Regarding the preference of the birds for TM meal in a scale of daily intake, in which the amount of consumption means high or low preference for the ingredient, it is possible to verify that the vectors for days 25 to 32 of birds’ age point in the direction of the most preferred ingredient, TM meal, in four out of six circles (replicates) of the test group. In the graph, bird preference increases as the vectors move in a positive direction from the origin to the arrow. This finding evidences the higher consumption of TM meal compared to the other components in the last days of the trial. In contrast, the graph allows us to infer that at the beginning of the trial (from days 15–19 of age), ground corn was the most preferred ingredient. From days 20 to 24 of age there was no clear preference, indicating the period of shift between ground corn and TM meal. Extruded semi-whole soybean meal and supplement mixture were the least preferred ingredients by the birds during the experiment.

Data for growth performance are summarized in Table 7. Feed intake of the balanced complete diet and weight gain of the birds of C group in the three evaluation periods were higher than in the test group (*p* < 0.02), in which the birds had the choice of ingredients. For feed conversion ratio, no difference was observed between birds of the C group (1.76) and those of the test group (2.15) in the period from days 15 to 21 (*p* = 0.418). Interestingly, the feed conversions from days 22 to 28 of age were statistically different, with average values of 1.22 for the TM group vs. 1.59 for the C group (*p* = 0.004), and the same trend was observed from days 29 to 32, in which the feed conversion of the test group (1.36) was better than that of the control group (1.63, *p* = 0.014).

## 4. Discussion

The data on nutrient composition of TM larvae meal indicate that protein value is similar to those found in other studies showing that insects are a good source this nutrient; in particular, *T. molitor* has an average protein content of 526 g/kg DM [4,17,28,29]. Moreover, Finke [16] mentioned that *T. molitor* has a sufficient amount of protein for the growth of rats and chickens, being nutritionally equivalent to fish meal and soybean meal. Regarding the composition of essential amino acids, the TM meal used in this study showed higher values for valine and histidine compared to animal protein sources utilized in the feed industry, e.g., meat meal (24.5 and 9.5 g/kg DM, respectively) and fish meal (28.2 and 11.2 g/kg DM, respectively). In addition, it has similar or slightly higher contents of all amino acids compared to vegetable protein sources [5]. For Bukkens [30], in most cases, insect protein is better balanced than that of plants.

Differences observed between values of amino acids in the literature and the present study appear as a consequence of a wide variation in composition for TM meal from different databases. The variable content of amino acids may be due to factors such as methodology employed, local food availability, and larval stage [4,31,32]. Overall, our results are in agreement with those reported by Ravzanaadii et al. [26], evaluating the nutritional value of *T. molitor* as a food and feed source, as well as De Marco et al. [17] and Elahi et al. [33], who evaluated the potential use of *T. molitor* for broiler chickens.

For mineral composition, the analyzed values are within the range found in the literature. The concentrations of calcium and phosphorus are much lower than those in the usual ingredients of animal origin used in feeds, because insects have a soft structural body, not including bones. Phosphorus concentration is similar to that of soybean meal, but it is considered totally available [16,34]. Moreover, TM meal seems to be a very good source of trace minerals such as copper, iron, manganese, and zinc, in agreement with data reported by Finke [16]. These trace minerals are essential for biochemical processes in the body, participating actively in metabolic and immune responses for production [3]. According to Rumpold and Schluter [35], regarding the amount of zinc and iron for nutritional requirements, edible insects could be considered a food mineral supplement as they normally have high content of these minerals compared to animal protein sources.

Insect larvae meal is a rich source of energy due to its high fat content. Insects have a very relevant plasticity to modulate body fat composition. The main factor influencing it is the substrate in which the larvae are grown [36,37].

For fatty acid composition, it was observed that TM meal has a significant amount of palmitic, oleic, linoleic, and α-linolenic acid. Despite the variation in composition of fatty acids, these data are in close agreement with prior reports [7,26,35]. Unsaturated fatty acids seem to have biological importance as functional nutrients, modulating health effects in humans [38]. As TM meal shows good amounts of unsaturated fatty acids, it opens other possible applications to this novel alternative feed ingredient.

Measuring daily average feed intake, it was observed that in the first few days a wide variation in consumption occurred in the four components of all pens in the test group, indicating a peculiar feeding behavior of the birds. In addition, corn was the ingredient consumed in greatest amount by the birds (up to day 17 of age). From day 18 to day 24, there was a shift in this trend, showing that, among the protein ingredients, there was a preference for the TM meal compared to extruded semi-whole soybean meal. Starting at 25 days of age the intake of TM meal was superior compared to all other components, which evidences the acceptability and choice of this ingredient by the birds. Along the trial, the intake of TM meal in the test group increased considerably, reaching 34% of total consumption during the first seven days of experiment, 62% during the following seven days and, in the last four days, 58%. This preference may be based on the food habit of birds, once they have the practice of entomophagy [3]. Moreover, the birds’ intense craving for TM meal might be also related to its nutritional composition (high energy and protein values) and may be associated with other undetermined properties.

Through a parametric evaluation of linear relationship for feed intake of all ingredients between the ages of the birds at 23, 25, 27, 29, and 30 days of age, it is possible to assume, based on very high positive correlations, that birds started to reach a constant balance of ingredient intake from the 11th day of the experiment, which refers to day 25 of bird age. The multidimensional preference analysis corroborates the explanation about the uniformity for consumption of the ingredients by the birds of test group from the 11th day of experimentation (day 25 of bird age). Once TM meal preference was established, the birds had also adapted to the choice of the other components, which reduced the variation of intake among them, as can be seen in Table 6. To the best of the authors’ knowledge, there are no data reported in the literature evaluating feed preference of birds for insect meal to be compared to the findings described.

In the present study, the data for feed intake showed a difference in consumption up to 30% between groups. During the first two days of the experiment, the diet of the test group was clearly unbalanced (Table 5), with the chickens consuming 80–90% corn; in addition, during the first week, total feed intake and weight gain of the test group were reduced by 33–35%. The low initial weight gain due to the unbalanced diet may have been harmful for further growth of the chickens. According to Yo et al. [39], sensory factors (e.g., color) play an important role in ingredient intake and regulation; thus, birds fed a basal diet (corn–soybean meal) before the initiation of the trial might have found ground corn similar to it, unleashing a preference for this component. Along the days, birds instigated by their active and curious behavior were able to self-select other components to promote regular growth. Interestingly, the most consumed component reverted to be TM meal (protein- and energy-rich), followed by ground corn (energy content), which indicates the capability of birds to regulate the consumption of ingredients to maintain the energy: protein ratio according to their nutritional needs [40]. Moreover, the unquestionable shift from a conventional protein source (soybean) to TM meal was observed and it may demonstrate that birds opted to feed on TM meal because of its sensory characteristics as well as good nutritional profile. Accordingly, Biasato et al. [41] suggested that improved diet palatability might be responsible for the increased feed intake and weight gain when chickens were fed TM meal in their study.

Although birds of the test group had the capacity to balance their consumption as the trial advanced, weight gain was also proportionally lower compared to the C group. This can be explained by the cafeteria-type feeding system (free-choice), as during a period of time (days 15–25 of age) birds were trying to adjust an appropriate diet to meet daily requirements [42], which affected the weight gain of the test group. It must be noted that measurable TM meal intake took up to 10 days in some pens and its consumption was immediate in other pens (data not shown). This fact resulted in great differences among pens for nutrient intake and expected differences in feed efficiency. Therefore, the values of feed conversion encountered may be impaired, especially in the period from days 15 to 21. Once birds of the TM group started to better balance their diets, it was possible to verify a great improvement in feed conversion from days 22 to 28 and days 29 to 32 of age, in which this variable was better for the test group compared to the control group. Even though it is known that a balanced complete diet supplies an adequate mixture of all nutrients required to improve efficiency [43], this current study shows interesting features about TM meal in its capacity to improve feed conversion in a free-choice feeding trial. It is possible to notice that as birds were adapting to the free-choice diet, they started to recover in performance continuously.

The present findings about feed preference with TM meal are the first data available, which might aggregate information in the literature to indicate that birds have a great preference for insect meal-based products. New studies must be done in order to gather data on the digestibility, performance, and immune system of birds fed insect meal for future global applications as a feed.

## 5. Conclusions

Chickens exhibited a preference for *Tenebrio molitor* meal, resulting in improved feed efficiency, which allows to conclude that it can be a suitable feed alternative for poultry.

## Figures and Tables

**Table 1:** Composition of the standard diet of control group, as fed basis.

Ingredients (g/kg, Unless Noted)	14–32 Days
Corn	544.5
Extruded semi-whole soybean 41.7% CP	422.0
Dicalcium phosphate	14.1
Limestone	7.9
Salt	5.0
DL-Methionine	2.5
Vitamin premix ^1^	1.2
L-Lysine 77%	1.1
Choline chloride 70%	0.6
Salinomycin 12%	0.6
Mineral premix ^2^	0.5
Total	1000
Nutrient profile ^3^	
Crude protein	233.4
Ether extract	60.9
Crude fiber	24.8
Available phosphorus	3.7
Calcium	7.6
Methionine	5.4
Lysine	11.2
Methionine + Cysteine	8.3
Threonine	7.5
AMEn (MJ/kg)	12.71

^1^ DSM Nutritional Products, Composition per kg of diet: Vit. A—10,800 UI; Vit. D3—3000 UI; Vit. E—24 UI; Vit. K3—3 mg; Vit. B1—2.4 mg; Vit. B2—7.2 mg; Vit. B6—3.6 mg; Vit. B12—18 μg; Nicotinic acid—42 mg; Pantothenic acid—21.6 mg; Biotin—0.12 mg; Folic acid—1.8 mg; Selenium—0.3 mg. ^2^ DSM Nutritional Products, Composition per kg of diet: Manganese—80 mg; Iron—50 mg; Zinc—50 mg; Copper—10 mg; Cobalt—1 mg; Iodine—1 mg. ^3^ On a 88.9% dry matter basis, the crude protein, ether extract, and crude fiber are analyzed values, others are calculated values.

**Table 2:** Chemical composition and mineral content of *T. molitor* (TM) meal used in the study compared to range values in the literature (dry matter basis).

	TM Meal	Literature ^1^
Dry matter (g/kg)	936.7	946.7–962.8
Crude protein (g/kg)	521.0	492.0–555.8
Gross energy (MJ/kg)	28.45	24.40–32.42
Ash (g/kg)	41.2	28.6–31.0
Ether extract (g/kg)	317.4	280.0–361.0
Calcium (mg/kg)	1228	169–2700
Phosphorus (mg/kg)	6058	2850–7800
Cu (mg/kg)	6.8	6.1–16.0
Fe (mg/kg)	62.4	20.6–66.9
Mn (mg/kg)	12.9	5.2–9.0
Zn (mg/kg)	115.1	52.0–116.0

^1^ References: [4,5,7,16,17,26,27,28].

**Table 3:** Amino acid profile of *T. molitor* (TM) meal used in the study compared to range values in the literature (g/kg of dry matter basis). AA: amino acid composition; DM: dry matter.

	TM Meal	Literature ^1^
**Indispensable AA (g/kg of DM)**		
Arginine	28.2	23.6–34.5
Histidine	17.5	14.2–20.1
Isoleucine	22.5	21.0–35.6
Leucine	38.0	31.5–45.8
Lysine	30.0	25.7–35.9
Methionine	7.4	6.3–10.1
Methionine + Cysteine	12.2	9.4–22.6
Phenylalanine	23.9	16.1–23.0
Threonine	20.2	18.1–26.1
Valine	32.5	24.4–39.7
**Dispensable AA (g/kg of DM)**		
Alanine	38.0	36.8–44.3
Aspartic acid	44.2	35.9–50.5
Cysteine	4.8	3.1–12.5
Glycine	27.0	22.1–31.8
Glutamic acid	62.9	56.8–79.7
Proline	30.9	30.2–43.4
Serine	23.3	20.9–37.0
Tyrosine	45.9	28.4–39.1

^1^ References: [7,17,26,27,28].

**Table 4:** Fatty acid content of *T. molitor* (TM) meal used in the study compared to the literature (g/100 g of EE).

Fatty Acid	TM Meal	Literature ^1^
Myristic acid (C14:0)	3.1	2.9–4.0
Palmitic acid (C16:0)	15.4	16.7–22.9
Stearic acid (C18:0)	2.3	2.5–3.9
Oleic acid (C18:1)	45.3	37.7–53.9
Linoleic acid (C18:2n6)	26.2	27.4–34.8
α-Linolenic acid (18:3n3)	1.1	1.3–1.4

^1^ References: [4,5,16,26,28].

**Table 5:** Daily consumption of ground corn, extruded semi-whole soybean, *T. molitor* (TM) meal, and supplement mixture of the test group from day 15 to day 32, data in grams per pen (five chickens) ± standard deviation.

Days	Corn	Extruded s-w Soybean	TM Meal	Supplement Mixture	*p* Value
**D15**	250 ± 31 ^a^	12 ± 16 ^b^	8 ± 2 ^b^	3 ± 3 ^b^	<0.0001
**D16**	284 ± 35 ^a^	35 ± 29 ^b^	19 ± 27 ^b^	14 ± 13 ^b^	<0.0001
**D17**	254 ± 128 ^a^	21 ± 32 ^b^	102 ± 126 ^b^	21 ± 28 ^b^	<0.001
**D18**	225 ± 118 ^a^	19 ± 27 ^c^	207 ± 180 ^ab^	31 ± 19 ^bc^	<0.005
**D19**	174 ± 157 ^a^	18 ± 27 ^a^	193 ± 147 ^a^	24 ± 22 ^a^	<0.05
**D20**	136 ± 106 ^ab^	33 ± 62 ^b^	209 ± 163 ^a^	21 ± 13 ^b^	<0.05
**D21**	138 ± 118 ^ab^	37 ± 76 ^b^	268 ± 129 ^a^	22 ± 16 ^b^	<0.001
**D22**	116 ± 108 ^ab^	37 ± 87 ^b^	270 ± 135 ^a^	19 ± 12 ^b^	<0.001
**D23**	148 ± 104 ^ab^	31 ± 68 ^b^	240 ± 120 ^a^	20 ± 18 ^b^	<0.001
**D24**	139 ± 77 ^ab^	35 ± 83 ^b^	239 ± 98 ^a^	29 ± 25 ^b^	<0.001
**D25**	147 ± 49 ^b^	14 ± 17 ^c^	307 ± 43 ^a^	26 ± 15 ^c^	<0.0001
**D26**	133 ± 69 ^b^	18 ± 18 ^c^	312 ± 37 ^a^	42 ± 22 ^c^	<0.0001
**D27**	135 ± 64 ^b^	7 ± 6 ^c^	296 ± 42 ^a^	59 ± 59 ^bc^	<0.0001
**D28**	143 ± 68 ^b^	12 ± 7 ^c^	336 ± 48 ^a^	48 ± 19 ^c^	<0.0001
**D29**	148 ± 74 ^b^	12 ± 7 ^c^	339 ± 41 ^a^	37 ± 13 ^c^	<0.0001
**D30**	173 ± 76 ^b^	20 ± 24 ^c^	296 ± 34 ^a^	37 ± 13 ^c^	<0.0001
**D31**	196 ± 72 ^b^	9 ± 9 ^c^	293 ± 61 ^a^	38 ± 15 ^c^	<0.0001
**D32**	210 ± 90 ^b^	20 ± 19 ^c^	308 ± 55 ^a^	31 ± 8 ^c^	<0.0001

^a,b,c^ Mean values within a row having different superscripts are statistically different by Tukey test (*p* < 0.05).

**Table 6:** Pearson’s correlation coefficient and *p*-value of feed intake of all ingredients between the ages of the birds at 23, 25, 27, 29, and 30 days of age.

Intake of Feed Components	Correlation Coefficient (r)	*p* Value
d23 vs. d25	0.71817	<0.0001
d23 vs. d27	0.72751	<0.0001
d23 vs. d29	0.68919	0.0002
d23 vs. d30	0.71423	<0.0001
d25 vs. d27	0.93712	<0.0001
d25 vs. d29	0.96498	<0.0001
d25 vs. d30	0.95711	<0.0001
d27 vs. d29	0.95661	<0.0001
d27 vs. d30	0.93542	<0.0001
d29 vs. d30	0.98022	<0.0001

**Table 7:** Feed intake, weight gain, and feed conversion ratio of the C group and TM group per period (average/bird ± standard deviation).

Variables	Treatments ^1^		*p*-Value
	C	TM	
Days 15–21			
Feed Intake (g)	799 ^a^ ± 67	537 ^b^ ± 54	<0.0001
Weight Gain (g)	455 ^a^ ± 11	297 ^b^ ± 111	0.006
Feed Conversion Ratio	1.76 ^a^ ± 0.14	2.15 ^a^ ± 1.14	0.418
Days 22–28			
Feed Intake (g)	966 ^a^ ± 72	638 ^b^ ± 77	<0.0001
Weight Gain (g)	611 ^a^ ± 37	528 ^b^ ± 60	0.016
Feed Conversion Ratio	1.59 ^a^ ± 0.16	1.22 ^b^ ± 0.18	0.004
Days 29–32			
Feed Intake (g)	699 ^a^ ± 60	414 ^b^ ± 68	<0.0001
Weight Gain (g)	430 ^a^ ± 37	311 ^b^ ± 72	0.005
Feed Conversion Ratio	1.63 ^a^ ± 0.07	1.36 ^b^ ± 0.22	0.014

^1^ Treatments: C = Control group; TM = *T. molitor* group. ^a,b^ Mean values within a row having different superscripts are statistically different by the *t* test (*p* < 0.05).
